# Evolution of Copper Transporting ATPases in Eukaryotic Organisms

**DOI:** 10.2174/138920212799860661

**Published:** 2012-04

**Authors:** Arnab Gupta, Svetlana Lutsenko

**Affiliations:** Department of Physiology, Johns Hopkins University, Baltimore, MD 21205, USA

**Keywords:** ATPase, ATP7B, Copper, CopA.

## Abstract

Copper is an essential nutrient for most life forms, however in excess it can be harmful. The ATP-driven copper pumps (Copper-ATPases) play critical role in living organisms by maintaining appropriate copper levels in cells and tissues. These evolutionary conserved polytopic membrane proteins are present in all phyla from simplest life forms (bacteria) to highly evolved eukaryotes (Homo sapiens). The presumed early function in metal detoxification remains the main function of Copper-ATPases in prokaryotic kingdom. In eukaryotes, in addition to removing excess copper from the cell, Copper-ATPases have another equally important function - to supply copper to copper dependent enzymes within the secretory pathway. This review focuses on the origin and diversification of Copper ATPases in eukaryotic organisms. From a single Copper ATPase in protozoans, a divergence into two functionally distinct ATPases is observed with the evolutionary appearance of chordates. Among the key functional domains of Copper-ATPases, the metal-binding N-terminal domain could be responsible for functional diversification of the copper ATPases during the course of evolution.

## ORIGIN OF COPPER ATPases

Copper-containing proteins appeared early in evolution, likely in response to increasing need to utilize oxygen and oxygen containing molecules. The abundance and biologic availability of metals through geological periods along with solubility of their sulphide salts are thought to have influenced the appearance and evolution of metalloproteins. In an ancient ocean, the metals existed mostly as sulfides with the following relative solubility: Fe>Mn, Ni, Co>>Cd, Zn, Cu [1]. Gradual oxygenation of the atmosphere changed the redox state of iron (Fe^2+^ to Fe^3+^), which reduced its solubility and hence, availability. At the same time, the levels of bioavailable copper increased [1]. Ocean oxygenation caused a shift from its sulfide towards a more soluble sulfate form. Copper was eventually incorporated into enzymes, the important function of which was to utilize oxygen for respiration (cytochrome c oxidase), photosynthesis (plastocyanin) and to protect cells against byproducts of oxygen utilization (such as superoxide and peroxide). Employing copper in biology necessitated the development of molecular machinery for the delivery, distribution, and export of copper from the cell.

Recent studies suggest that the utilization of oxygen and copper by early prokaryotes followed parallel evolution. Comparative genome analysis of copper transporters and cuproproteomes has found that most aerobic prokaryotes employ copper for their physiologic chemistry, whereas anaerobes do not [2]. *In vitro*, copper can bind to various synthetic and biological molecules (amino-acids, nucleotides, phospholipids); however, in cells copper is used and handled mostly by proteins, which employ this metal as a cofactor to maintain their structure or function. In addition to cuproenzymes, there are four classes of proteins that regulate copper levels in a cell without using copper for enzymatic reactions. In eukaryotic cells, such proteins can be divided into membrane bound importers, ATP-driven copper exporters, the soluble copper-delivery molecules, or copper-chaperones, and intracellular copper chelators (metallo-thioneins). It has been hypothesized that in evolution copper exporters appeared prior to copper chaperones or copper importers [3]. 

The discovery of microfossils in deep sea volcanic rocks led to the proposal that the first life on Earth appeared ~3.2 billion years ago [4]. These vents release metals like iron, zinc and copper from the earth’s substratum, and the hot and acidic water further facilitates this leaching process. To survive harsh conditions of hydrothermal vents, the primordial organisms needed special protective mechanisms. It is conceivable that the evolution of metal exporters provided early organisms with the resistance mechanisms against excess metals. 

The Copper-ATPases (or ATP driven copper pumps) play the major role in maintaining copper balance in cells. Copper-ATPases have been studied in significant detail in bacteria and archea, and several recent reviews provide comprehensive summary of this work [3,5,6]. By comparison, Copper-ATPases in single cellular eukaryotes, non-chordates and lower chordates are much less characterized. This review focuses mainly on eukaryotic Copper ATPases, including protists, yeasts, and animals. We will provide a brief introduction of bacterial Copper-ATPases, since this information helps to understand the functioning of these proteins in the organisms, which appeared later in the evolution.

## BACTERIA AND ARCHEA ILLUSTRATE EVOLUTIONARY CONSERVATION OF COPPER-ATPase

*Archeoglobus fulgidus* is one of the phylogenetically primitive organisms, in which copper transporters have been studied in detail. *A. fulgidus* is the sulphur-metabolising archeobacteria and a hyperthermophile found in hydrothermal vents, oil deposits, and hot springs. Copper ATPases (CopA and CopB) in this organism contain the same functional domains as Copper-ATPases of eukaryotes (see details below); however, they show maximum activity at 75–85°C [7]. Interestingly, the presence of two Copper ATPases might be needed to accommodate different chemical properties of Cu+ and Cu++ ions, since CopA and CopB, while homologous, have distinct metal specificity and structural differences in regions associated with metal binding. In *A. fulgidus*, CopA is activated by Ag^+^>Cu^+^ whereas CopB is activated by Cu^2+^>Ag^+^>Cu^+ ^[7]. 

CopA and CopB were also identified and studied in mesophilic bacteria, *Enterococcus hirae *[8-11]. Similarly to *A. fulgidus*, these two transporters show high sequence homology to each other except in regions associated with metal binding and to Copper-ATPases of higher organisms (human, mouse etc). Disruption of *CopB* or both *CopB* and *CopA* drastically decreases resistance of the bacteria to high copper. In contrast, bacteria lacking only *CopA* ceased to grow after a few generations in media lacking copper, whereas normal growth was observed in wt cells. It is thought that CopA facilitates copper acquisition under copper limiting conditions, whereas CopB extrudes excess copper. The physiologic need for copper uptake system is not entirely clear, as in these gram-positive bacteria no copper dependent cytoplasmic enzymes are known [3] However, it can be speculated that CopA in these bacteria exports copper to the periplasmic space where it binds to the cuproproteins such as multicopper oxidases. In this scenario, the decreased growth phenotype observed in bacteria lacking CopA could be due to reduced copper availability to the cuproproteins of the periplasm. The presence in *E. hirae* of a soluble copper-chaperone CopZ and a copper-dependent transcription factor CopY [12,13] may also indicate that CopA is a component of a regulatory network that provides copper to the regulator molecules in a biologically available form. 

The original function of Copper-ATPases, the maintenance of proper intracellular concentration of copper and protection against metal toxicity, has been preserved throughout evolution. In high eukaryotes, it has been adopted to provide a vectorial transfer of copper through complex barriers (in such processes as the dietary copper absorption *via *intestine or the delivery of copper into the brain through a blood brain barrier). In addition to the export function, new roles for Copper ATPase appeared and were retained in evolution. An opportunistic human pathogen, *Pseudomonas aeruginosa, *which survives in the atmosphere as well as in hypoxic conditions of human body, has two Copper-ATPases (CopA1 and CopA2). Gonzalez-Guerrero and colleagues demonstrated that these two structurally similar Copper-ATPases are both vital for virulence, but have distinct functions [14]. Expressed in response to high copper, CopA1 mainly functions as a copper exporter and provides resistance to the metal, as evidenced by the inability of CopA1 deletion mutants to grow in high copper. CopA2, on the other hand, is expressed in association with the subunits of cytochrome oxidase. Mutations in CopA2 lead to higher oxidative stress in response to H_2_O_2 _and reduced cytochrome c oxidase activity, whereas no alteration in copper tolerance is observed [14]. Interestingly, the expression of the Copper-ATPase genes is higher (>150 fold for CopA1 and ~75 fold for CopA2) in bacteria that infects A. thaliana leaves compared to bacteria grown in LB media, emphasizing their role in pathogenicity. 

The possible role for CopA1 during infection could be to protect the bacterium against excess copper present in the phagosome of macrophages [15]. CopA2 participates in the synthesis of cytochrome *c *oxidase, which is required for normal metabolism of bacterium. A similar observation of the importance of copper transporters for bacterial virulence was made for a facultative anaerobic bacteria *Staphylococcus aureus*. Screening for genes induced in *S. aureus *during infection identified a gene *ivi44*, that was critical for virulence and it encoded a protein with 50% sequence identity to CopA of *E. coli *[16]. These observations suggest that during evolution the copper transporters function was adopted to provide competitive advantage during pathogenesis and host infection by the bacteria.

## COPPER ATPases USE THE SAME TRANSPORT MECHANISM IN DIFFERENT CELLULAR LOCATIONS 

Copper-ATPases are polytopic membrane proteins with essential roles in all phyla. In prokaryotes, Copper-ATPases function at the plasma membrane, whereas in eukaryotes these proteins are often found in the intracellular compartments, where they transport copper from the cytosol into the lumen of the secretory pathway using the energy derived from ATP hydrolysis. In lower eukaryotes, i.e. the non-chordates, a single Copper-ATPase (commonly referred as ATP7 or simply CuATPase) is present. With a growing complexity of organisms (in chordates), ATP7 branched out as ATP7A and ATP7B. These homologues, though sharing significant sequence similarity, have different expression in tissues, unique functions and distinct intracellular trafficking properties [17]. 

In a eukaryotic cell, under normal conditions, Copper-ATPases may localize primarily to the Golgi membranes, intracellular vesicles, or the plasma membrane. The subcellular localization of Copper-ATPases correlates with their primary function in the cell, such as sequestration of excess copper for efflux (homeostatic function) or copper transfer to copper binding proteins for functional maturation (biosynthetic function) [17]. In yeast *Saccharomyeces cerevisiae*, the Copper-ATPase Ccc2p permanently resides in the Golgi network, where it transfers copper to Fet3p, a copper-dependent ferroxidase [18]. The copper export function in yeast is accomplished by a separate ATPase (see below). In contrast, the mammalian Copper-ATPases (ATP7B in liver cells and ATP7A in most cells) have dual function and dual localization. One location is the *trans*-Golgi network where these transporters deliver copper into the lumen of secretory pathway for biosynthetic incorporation into copper-dependent enzymes [17,19-24]. To facilitate copper export from the cell (under conditions of excess copper) ATP7A and ATP7B traffic from the Golgi network to their second location - vesicles that eventually fuse with the plasma membrane [25-27]. It is still not well understood at what point in the evolution, Copper-ATPase converted from a non-trafficking protein (as in yeast) with permanent sub-cellular localization to a trafficking form (as in chordates), which localizes to more than one sub-cellular organelle depending on intracellular copper levels. 

The ATPase-driven copper transport is a multi-step process that involves the acquisition of copper from the cytosol, the delivery of cytosolic copper to the intramembraneous site of the transporter and a subsequent release of the metal at the luminal side of the membrane. Copper release occurs in synchrony to the binding and hydrolysis of ATP by the protein. The Copper-ATPases are members of a large family of P-type ATPases. The characteristic step in their catalytic cycle is the transfer of ɣ-phosphate of ATP to the invariant Asp residue in the DKTG motif with the formation of a transient phosphorylated intermediate [17]. This reaction can be monitored *in vitro* using membrane preparations containing Copper-ATPase [28]. For the analysis of Copper-ATPase transport function, yeast complementation assay has proven to be convenient and informative [29]. This assay utilizes a mutant yeast strain in which the activity of endogenous Copper ATPase, Ccc2p is disrupted by gene deletion. As a result, copper is not delivered into the secretory pathway to a copper-dependent ferroxidase Fet3p, which normally functions with the high-affinity iron transporter Ftr1p to import iron [30,31]. In the absence of Fet3p activity, cells become incapable of high-affinity iron uptake [32] and the Δ*ccc2* cells do not grow on iron-limited medium; expressing the active Copper-ATPase restores copper transport to Fet3 and cell viability [29].

## MODULAR STRUCTURE OF EUKARYOTIC COPPER ATPase SUGGESTS DIFFERENT MODES OF REGULATION OF THEIR COMMON FUNCTION

Copper-ATPases from bacterial to humans share the same domain disposition. Although, the structure of eukaryotic Copper-ATPases is not yet available, the structure of prokaryotic CopA of *Legionella pneumophila* (LCopA) has recently been solved and yielded important insights into the molecular architecture of this class of proteins [33]. LCopA is 736 amino-acids long, whereas the length of eukaryotic Copper-ATPases varies between ~1000 amino acids (Ccc2p in Saccharomyeces cerevisiae) to 1500 amino acids (human ATP7A). The difference in length is largely due to a significant variation of the N- and C-termini, as well as presence of functionally important sequence inserts throughout the molecule (see below). The LCopA structure is essentially a core common for the prokaryotic and eukaryotic Copper-ATPases.

The domain composition of the core and the overall fold of individual domains are highly conserved. The Copper-ATPases have eight trans-membrane segments (TMS) with the N- and C-termini of the protein both oriented towards the cytosol. The cytosolic portion of the Copper-ATPases contains several functional domains: the N-terminal copper-binding domain, the actuator (A-domain), the ATP binding domain (which includes the nucleotide binding (or N-) domain, and the phosphorylation (or P) - domain, and the C-terminus [34]. The N-terminal copper-binding domain contains one to six sub-domains that each has a ferredoxin-like fold (βαββαβ). Each sub-domain also contains a single copper-binding site GMxCxxC, in which two invariant cysteines of the CxxC motif coordinate Cu+1 [17,35,36]. Fig. (**1a**) illustrates the domain organization of the human copper transporter ATP7B. 

**Fig. (1):**
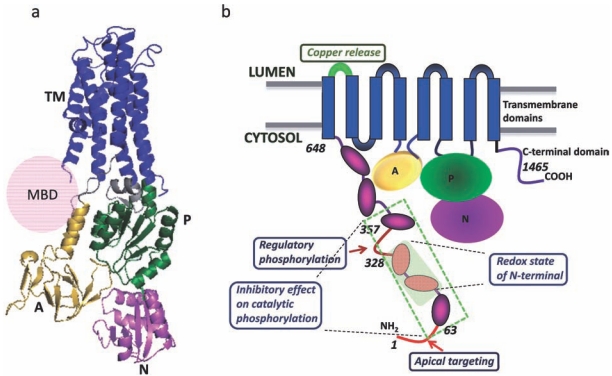
Structural organization of human ATP7B. (**a**) Three dimensional structure of ATP7B showing the transmembrane domains (blue), actuator domain (gold), nucleotide binding domain (pink), phosphorylation domain (green). (**b**) The regulatory sites which are important for regulation and localization of the protein have been shown. The N-terminal domain (1-648) is the major site for regulation of the molecule. The luminal loop connecting TM1 and 2 (shown in green) is important for copper binding and release (ATP7A).

Sequence comparison of functional domains in evolutionarily distinct organisms demonstrates very different evolutionary trends for these domains. The P-domain (that houses the catalytic aspartate) is most conserved, both with respect to its sequence and size. Similarly, high conservation and little variation in size are found for the A-domain, which plays the central role in protein dephosphorylation and conformational transitions [37-39]. In contrast, two other key functional domains: the transmembrane portion and the N-domain show significant sequence variation as well as some variation in size. Mapping conserved residues to the transmembrane portion illustrates that these residues face inwards most likely forming the copper-translocation pathway [33]. More exterior transmembrane domains and the residues facing lipids are not conserved, as would be expected given that lipid environment may vary greatly between the organisms. The size of the transmembrane domain is essentially unchanged from prokaryotes to mammals, with a notable exception of a sequence insert located between TM1 and TM2 in human ATP7A. Recent studies indicate that this insert binds copper and is important in regulation of copper release into the lumen (see Fig. **1b**) [40].

The N-domain organization is very characteristic and shows interesting progression in evolution. There is a core structure with a characteristic Rossman fold that is unchanged from bacterial to humans. In addition, all eukaryotic N-domains have a distinct sequence insert between the β strand 3 and 4 [41], the length of which generally increases with evolution of the ATPases. In yeast Ccc2p, the insert is very short and the N-domain is very similar to that of bacterial ATPases. In *Caenorhabditis elegans*, *Xenopus laevis *and higher eukaryotes, the length of the insert increases, reaching about 30% of the entire N-domain sequence in human ATP7A. Interestingly, the size of insert in ATP7A across species tends to be bigger than that of ATP7B irrespective of their evolutionary position, e.g. the insert in the N-domain of Zebrafish ATP7A is longer than the insert in human ATP7B N-domain. Functional studies indicate that the additional sequence present in the insert is not important for binding of ATP (at least in human enzymes – [41]), suggesting that this sizable addition to the N-domain may play an important role in regulation of Copper-ATPase’ activity and/or localization. 

The highest sequence variability and increased complexity of overall design are found in the N-terminal metal-binding domain of Copper-ATPases. There is a significant evidence for the complex regulatory role of this region, particularly in eukaryotic Copper-ATPases, which we will discuss in detail below. Fig. (**1b**) illustrates that in human ATPases more than 50% of protein structure is employed in regulation of the transporter.

## PHYLOGRAM ANALYSIS SHOWS HIGHER CONSERVATION AMONG ORTHOLOGS COMPARED TO HOMOLOGUES

**Fig. (2):**
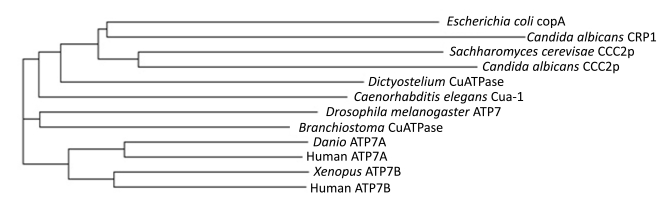
Phylogram of the copper ATPases of species belonging to different evolutionary positions.

Phylogram analysis of Copper-ATPases in representative organisms from different groups (Bacteria (*E.coli*) CopA, Protista (*Dictyostelium*), Yeast (*Candida* & *Saccharo-myeces*) Nematoda (*Cenorhabditis*), Arthropoda (*Droso-phila*), Cephalochordata: (*Branchiostoma*, Amphioxus), Chordata (Pisces: *Danio*, Amphibia: *Xenopus*, Mammalia: *Homo sapiens*) illustrates (Fig. **2**) that yeast and bacterial Copper-ATPases branched out early in evolution and evolved independently. It is interesting that the ATP7A-like proteins in phyla with large evolutionary distances share higher homology between each other than ATP7A and ATP7B of the same organism (Fig. **2**). For example, human ATP7A has 56% identity to a human ATP7B, but 65% identity with *Danio rerio* ATP7A (GenBank: AAZ07896.1). Similarly, human ATP7B shared 66% homology with a predicted amphibian ATP7B (Xenopus; XP_002936778.1). This pattern suggests that the functional specialization of the two transporters appeared soon after the gene duplication event, which is thought to produce two Copper-ATPases in the same organism, and that the structural differences between 7A and 7B transporters are governed by their function and are not organism-specific. Similarly, in the phylogram of yeast Copper-ATPases, Ccc2p proteins of *Candida* and *Saccharomyeces* are closer to each other than *Candida’s* CaCRP1 and Ccc2p. Interestingly, *C. albicans *is estimated to be about 10^8^ years removed in evolution from *S. cerevisiae *[42]*. *During this period, as a result of adaptation to very different environments - an animal body (*C. albicans*) and rotting fruit (*S.* *cerevisiae*), *Candida* diverged by evolving a novel Copper-ATPases (CaCRP1) presumably from the already existing Ccc2p, however maintaining high homology with the common Copper-ATPase (Ccc2p).

## YEAST COPPER ATPases: TWO TRANSPORTERS WITH DISTINCT FUNCTIONS

How does an organism adapt to conditions where the copper-transport activity is required at distinct cellular locations: in the intracellular compartments for cuproenzyme biosynthesis and at the plasma membrane for cell protection against copper overload? Eukaryotic cells solve this problem in several ways. In the baker’s yeast, *Saccaharomyeces cerevisiae,* the copper ATPase Ccc2p is not involved in resistance to high copper and is located in membranes of Golgi. This cellular location is consistent with its primary role in the biosynthesis of copper-dependent enzymes. As described above, the biogenesis of the multicopper ferroxidase Fet3 occurs in a Golgi and requires functional Ccc2p [32]. Although Cccp2 is not involved in copper export across plasma membrane, *S. cerevisiae* is resistant to high copper and is able to grow in the medium with up to 2mM CuSO_4_ [42]. This is because a copper importer (Ctr1) rather than Ccc2p is responsible for regulating cellular levels of copper and metal resistance of *S. cerevisiae*. Specifically, in high copper, the uptake *via *Ctr1 is rapidly downregulated by decreasing the amount of this transporter at the plasma membrane [43]. 

The diploid opportunistic fungi,* C. albicans,* has additional mechanisms to maintain copper levels and is even more copper resistant, being able to grow in media containing 20mM CuSO_4_. In *C. albicans,* in addition to a Ccc2p orthologue (CaCCC2p), a second copper-transporting P-type ATPase, Ca*CRP1*, is expressed and contributes to high copper resistance [42]. Studies with the GFP-fusion of CaCrp1 revealed that this Copper-ATPase is located at the plasma membrane. Disruption of Ca*CRP1 *had a dramatic effect on organism sensitivity to copper. The minimum inhibitory concentration of copper for the Ca*crp1*∆ strain is reduced to 0.5 mM (compared to 25 mM for the wild-type), whereas resistance to Ag^+^, Cd^2+^ and Zn^2+^ remains unaltered. This observation suggests that CaCrp1 plays the major role in copper detoxification *via *copper efflux*. *

It appears that cellular pH homeostasis is linked to copper export. It has been observed that yeast sensitivity to copper markedly increases with decreasing pH of the growth media. The yeast becomes particularly sensitive at pH 3.0 and can only tolerate 25 µM copper in the medium, possibly due to due to inability of copper binding proteins to retain copper. The ∆CaCRP1 mutant is even more sensitive and fails to grow in 2uM Cu^+1^ as compared to 25 µM Cu^+1^ for the wt strain. As an opportunistic pathogen, *C. albicans *is found in the digestive tract of warm-blooded animals where the environment is largely anaerobic and predominantly acidic. Under these conditions even relatively low levels of copper found in food may become toxic to the yeast further emphasizing the physiologic importance of CaCRP1. 

In another study, the CRP1 gene of *Candida albicans* was designated as Crd1 [44]. Crd1 was also shown to be critical for copper efflux, but at variance with the above study Crd1 was found to transport silver. The authors proposed an interesting hypothesis regarding silver resistance due to Crd1. *C. albicans *is a frequent nosocomical infectious agent in burn patients and patients with catheters. Since silver was used as a microbiocidal agent for many years, and silver products are commonly used as a burn antiseptic, it was suggested that Crd1p may enhance the chances of *C albicans*’ survival as a nosocomical pathogen by increasing resistance to silver. 

## COPPER ATPase IN NON-FUNGI, LOWER EUKARYOTES: INDICATIONS OF COPPER DEPENDENT TRAFFICKING

Only limited information is available about Copper-ATPases in unicellular eukaryotes. Copper-ATPases have been studied in soil amoeba Dictyostelium and parasitic protozoan apicomplexa Cryptosporidium [45,46]. High resistance of Dictyostelia towards copper led to the identification of a copper-transporting ATPase [45]. Amoeba cells labeled with antibody against human Copper-ATPase ATP7A showed a diffused staining as well as bright spots, suggesting the presence of the transporter in different cell locations: the plasma membrane and internal ‘vacuoles’. Authors proposed that the presence of Copper-ATPase in localized spots as well as at the plasma membrane was indicative of constitutive trafficking of the transporter, necessary to conform high resistance to copper. Confirmation of anitibody specificity and the colocalization of the ATPase with organellar markers are necessary to verify this important conclusion. 

Similarly, in the apicomplexan pathogen, *Cryptosporidium parvum*, the immunostaining of Copper-ATPase, CpATPase2 [46] yielded both the diffuse and punctate staining leading to the conclusion that CpATPase2 within sporozoites localizes both at the plasma membrane and in the cytoplasmic ‘membranes’ (vesicles). If confirmed, the dual localization of the same Copper-ATPase in vesicles as well as the plasma membrane observed in amoeba is, probably, the earliest in evolution. The physiologic need for such dual localization can be explained by the fact that these organisms are exposed to variable and sometimes very high copper levels in the environment, i.e. to conditions when excess copper should be exported from the cell. At the same time, exposure to oxygen requires protective mechanisms at the cell surface in addition to copper extrusion. The protection of amoeba against oxygen-derived radicals at the cell surface is likely to be mediated by the extra-cellular superoxide-dismutase found in the genome. This copper-dependent enzyme receives its copper within the secretory pathway from Copper-ATPases (at least this is the case for high eukaryotes – [47]) and therefore intracellular copper transporter within the secretory pathway should be present. 

## COPPER ATPases IN METAZOANS: FROM A SINGLE TO TWO DISTINCT TRANSPORTERS

Information on the function and structure of Copper ATPases in lower metazoans are limited. A single copper ATPase (1238 amino acids), *CUA1, *containing three N-terminal copper binding sites has been cloned from the nematode worm, *Cenorhabditis elegans *[48]. Yeast complementation assay using the *CUA1* cDNA showed that CUA1 can rescue ∆ccc2p mutant of *S. cerevisiae* and hence it can function as a copper transporter. Due to limited studies, the role of CUA1 in normal physiological functions of the nematode is unknown. However, in *S. cerevisiae*, the HA-tagged CUA1 was shown to localize to large intracellular vesicles, possibly Golgi membranes, which can be further substantiated by the fact that, *CUA1* cDNA rescues ∆ccc2p mutant, as already mentioned.

More detailed information on the function of Copper ATPase in non-chordate metazoans is available for Drosophila. Drosophila posses a single Copper-ATPase DmATP7 (1254 amino acids) and has four metal binding GMxCxxC motifs in the N-terminal domain [49]. Though divergence of the single ATP7 into two Copper-ATPases (ATP7A and ATP7B as in mammals) was not observed in evolution before appearance of chordates, functional studies and sequence alignments provide a clue that DmATP7 is the precursor of ATP7A and not of ATP7B. This conclusion is consistent with the housekeeping function of ATP7A in organism homeostasis, and more specialized role of ATP7B [17].

Among the non-chordates Copper-ATPases, phenotypic studies had been carried out only on DmATP7 [49,50]. *DmATP7* mutants showed limited mobility as compared to the wt flies and had reduced pigmentation [49]. Norgate *et al. *have shown that *DmATP7 *participates in (a) delivering copper to cuproenzymes, such as tyrosinase that is required for pigmentation, (b) normal development of neuronal functions, and (c) removing excess cellular copper *via *facilitated efflux [49]. These observations have evolutionary significance since the mammalian ATP7A besides removing excess copper, also plays key roles in tyrosinase activation and normal neuronal functioning. Mammalian ATP7B on the other hand, is not involved in pigmentation and its role in neurogenesis is not well understood. 

Among early chordates, physiologic significance of Copper-ATPases was ascertained in zebrafish (*Danio rerio*). Copper chelation was found to disrupt the notochord and vascular development in this organism [51]. A Zebrafish with a mutated ATP7A gene (calamity; *Cal*) lacks melanin pigment and has a wavy notochord, mimicking the phenotypes induced by copper deficiency [52]. The Calamity phenotype is also similar in to human Menkes disease, where neuronal development of patients is severely impaired by an insufficient copper transport to the brain [53]. Expression of human ATP7A in the *Cal* mutants rescued the embryos, confirming the primary role of Copper-ATPase in the phenotype and functional conservation of ATP7A. Besides ATP7A, genomic sequence of zebrafish predicted a second copper transporter (XP_684415), which is thought to be an orthologue of human ATP7B. In this Copper-ATPase, only 4 copper binding sites were identified in the N-terminus, as compared to six in Zebrafish ATP7A. This may reflect functional difference between ATP7A and ATP7B, though further sequence verification and functional studies are needed to substantiate this conclusion.

Two distinct copper ATPases, ATP7A and ATP7B, are found in mammals [17]. They share high sequence homology and harbor six copper binding motifs in the N-terminus. In mammals, ATP7A has ubiquitous tissue distribution, whereas ATP7B is expressed most highly in the liver. It could be that the branching out of the primordial ATP7 into ATP7A and ATP7B coincided with the origin of liver in chordates. The two Copper-ATPases differ in their trafficking behavior in response to copper; i.e. in polarized cells ATP7A traffics to the basolateral membrane, whereas ATP7B relocalize to the apical membrane in response to high copper [20]. Studies of mammalian copper ATPases have been facilitated by the discovery of their roles in human diseases. Inactivation of ATP7A and ATP7B are associated with severe human disorders, Menkes disease and Wilson disease, respectively. Menkes disease is associated with severe developmental errors and neurological symptoms resulting from copper deficiency. Wilson disease on the other hand is a disease of copper accumulation in the liver and the brain leading to hepato-lenticular degeneration [17,23,29].

## THE N-TERMINAL OF COPPER-ATPases EVOLVED THE MOST

**Fig. (3):**
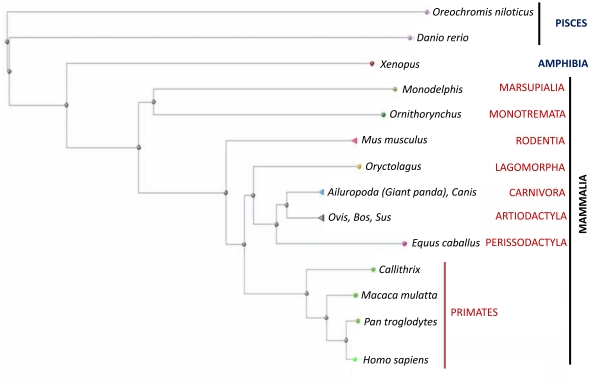
Phylogenetic tree of different species of chordates based upon the N-terminal of the copper ATPase ATP7B.

As described above, the transmembrane portion of the Copper-ATPase and the functional domains involved in ATP binding and hydrolysis are well conserved. In contrast, the N-terminal metal-binding domain seems to be the most altered during evolution. A general trend of increasing the number of copper binding sites is observed from lower to higher organisms. The *E. coli* Copper-ATPase CopA has two N-terminal metal binding domains (MBD), each with one copper-binding site. Also in the amoeba, Dictyostelium, only two MBD has been found. In yeast (Saccharomyeces and Candida), two MBDs are present. However, as mentioned earlier, CaCRP1 has three MBDs along with two proximal GMxCxxC motifs, which might have a role in copper binding and its plasma membrane localization. CUA1 of *C.elegans* has three MBDs. Further up the evolutionary ladder, Drosophila ATP7 has four MBDs. Among chordates (Danio, Xenopus, and humans) six MBDS are observed. Interestingly, in *Branchistoma* (amphioxus), in the hypothetical protein BRAFLDRAFT_63720, seven MBDS are detected. Phylogenetic analyses among the chordates using the N-terminal sequence of ATP7B revealed further interesting trend. The evolutionary position of the organism matched well with the evolutionary clustering and divergence of the N-terminal domain (Fig. **3**). This again points towards the fact that the evolutionary signatures are embedded within the N-termini of Copper-ATPases.

How can the N-terminal influence the properties of evolutionary different Copper-ATPase? Recent studies indicate that besides the number of MBDs (which may modulate the ATPase sensitivity to copper), the N-terminal domain is a subject of multiple regulatory activity. This increasingly multifaceted function of the N-terminus imparts uniqueness on the corresponding Copper-ATPases allowing them to adapt to a large spectrum of conditions.

## THE REGULATORY FUNCTIONS OF N-TERMINAL DOMAIN

Significant experimental evidence illustrates that the N-terminus is the major site of Copper-ATPase regulation (Fig. **1b**). The first four metal binding sites in MBD1–4 are present in chordate Cu-ATPases and have been reported to have a regulatory function in mammals [54,55]. Deletion of the region containing these four sites has no inhibitory effect on Copper-ATPases, instead it accelerates the catalytic cycle [54]. The deletion also does not alter the copper affinity of the intramembrane transport sites, at least in ATP7B [54]. This observation strongly suggests that the MBD1-4 region of the protein has a regulatory role and autoinhibits the catalytic function of the protein. The conclusion is supported by studies showing copper-dependent interactions between the N-terminal domain and the ATP-binding domain of ATP7B. Binding of copper to MBDs weakens the interactions with the ATP-binding domain [56]. 

Mutational analysis and the deletion studies were carried out by several laboratories to better understand the role of the multiple metal binding sites of the N-terminus [57]. Using the Golgi-enriched membrane fractions from CHO cells, it was found that all the MBDs in ATP7A could be mutated without complete loss of copper transport activity. But in another study, it was observed that two MBDs closest to the membrane (MBD5 and MBD6) were important for the functional activity of Copper ATPases. In ATP7A and ATP7B, at least one of these sites should be functional for normal copper transporting activity of the protein. Mutations of copper-coordinating cysteines in either MBD5 or MBD6 of ATP7B alter the apparent affinity of intramembrane binding site(s) for copper [54]. 

It has also been reported that the individual MBDs interact among themselves and this interaction(s) determine the redox state and copper binding efficiency of the N-terminal of ATP7B [58]. Mutating the MBDs-2 and 3 causes oxidation of cysteines in other MBDs and also loss of copper binding. In contrast, mutating MBD4 and MBD6 does not alter the redox status or copper binding of other sites. This suggest that MBD2 and MBD3 regulate copper binding to other metal-binding sites, whereas MBD4 and MBD6 receive copper independently, downstream of MBD2 and MBD3.

Copper binding and subsequent changes in domain-domain interactions most likely allow Copper-ATPases to adopt specific conformations that are critical for its targeting and trafficking. Besides the MBDs, the N-terminal also harbors structural elements that are critical for the directionality of Copper-ATPase trafficking in polarized epithelium. The very N-terminal of the protein has been implicated in determining the apical localization of the protein in high copper [59]. This stretch of N-terminal 63 amino acids (^1^Met-^63^Arg) is critical for the apical targeting of ATP7B. Deletion of the N-terminal sequence encompassing the first 4 or 5 MBDs and the terminal 63 amino acids (ΔMBD1–4 and ΔMBD1–5) resulted in protein being targeted constitutively to the basolateral surface. However, fusing the 1-63 N-terminal sequence to ATP7B with only MBD 5 and 6 restored the wild type behavior of the protein. Within the 1-63 sequence a stretch of nine amino acids, F_37_AFDNVGYE_45_, is an essential copper dependent apical targeting determinant [60]. This sequence is conserved in mammals and is partially conserved in lower chordates (e.g. in Xenopus, CAFDNRGY). This similarity suggests that apical trafficking behavior ATP7B may also be conserved in lower chordates.

Another mechanism by which the N-terminal domain may regulate the ATPase is through a kinase mediated phosphorylation. The N-terminus contains site(s) for kinase mediated phosphorylation; copper binding to ATP7B in cells increases protein phosphorylation and stimulates trafficking [61]. The recombinant N-terminal domain of ATP7B (N-ATP7B) is a target of phosphorylation *in vitro* and in cells. Analysis of phosphorylated peptides by mass spectrometry reveals that the loop connecting MBD3 and MBD4 contains phosphorylated residues. Interestingly, the region encompassing MBD1-3 (29 kDa fragments) was shown to have a inhibitory effect on phosphorylation [62]. In another study utilizing proteolysis and mass spectrometry, Ser(478) and Ser(481) in the loop between MBD 4 and 5 was found to be phosphorylated [63], suggesting that multiple phosphorylation sites may be present in the N-terminal domain and respond to different signaling stimuli. 

The phenomenon of regulatory phosphorylation of Copper ATPases is also observed in lower eukaryotes. The N-terminus of Ccc2p in yeast (Ser258) is a target of a kinase mediated phosphorylation, and this phosphorylation is higher in case of catalytically inactive protein containing mutated Asp residue (Asp627) [64]. The function of regulatory phosphorylation remains to be fully understood. As seen in yeast, the cross-talk between the two phosphorylation events (the catalytic phosphorylation of the P-domain and the regulatory phosphorylation in the N-terminus) may influence the activity and hence copper transport function of the protein. Additionally, regulatory phosphorylation might be critical for the trafficking of protein in response to copper. Evolutionary studies on regulatory phosphorylation, however, would not be possible until the phosphorylation residues are identified in the Copper ATPases.

In conclusion, the copper ATPases provides an interesting case to study evolution of a large and complex proteins, in parallel to the evolution of its harboring organism. Originating as a plasma membrane protein in thermophilic bacteria whose exclusive function was probably to export copper, Copper-ATPases have evolved in higher organisms as a transporter with more than one function and a very complex regulation. The increased functional complexity has been associated with structural changes. These changes did not affect equally all functional domains. Instead we show that that the N-terminus of the copper ATPases underwent the major diversification both structurally and functionally in comparison to other conserved domains.
